# Mesenchymal stem cells‐derived extracellular vesicles containing miR‐378a‐3p inhibit the occurrence of inflammatory bowel disease by targeting GATA2

**DOI:** 10.1111/jcmm.17176

**Published:** 2022-05-17

**Authors:** Ping Li, Hai‐Yan Zhang, Jian‐Zhen Gao, Wen‐Qiang Du, Dong Tang, Wei Wang, Liu‐Hua Wang

**Affiliations:** ^1^ Department of General Surgery Huaian Tumor Hospital & Huaian Hospital of Huaian City Huaian China; ^2^ Department of Central Laboratory Huaian Tumor Hospital & Huaian Hospital of Huaian City Huaian China; ^3^ Department of Experimental Surgery‐Cancer Metastasis Medical Faculty Mannheim Ruprecht Karls University Mannheim Germany; ^4^ Department of Clinical Nursing Huaian Tumor Hospital & Huaian Hospital of Huaian City Huaian China; ^5^ Department of General Surgery General Surgery Institute of Yangzhou Northern Jiangsu People's Hospital Clinical Medical College Yangzhou University Yangzhou China

**Keywords:** AQP4, GATA2, inflammatory bowel disease, miR‐378a‐3p, MSCs‐EVs, PPAR‐α

## Abstract

This study sought to determine whether mesenchymal stem cells‐derived extracellular vesicles (MSCs‐EVs) carrying microRNA‐378a‐3p (miR‐378a‐3p) could affect the pathogenesis of inflammatory bowel disease (IBD) by regulating the GATA‐binding protein 2 (GATA2)/aquaporin‐4 (AQP4)/peroxisome proliferator‐activated receptor α (PPAR‐α) axis. Initially, colon mucosa biopsy tissues were harvested from healthy controls and patients with IBD for qRT‐PCR and immunohistochemistry analysis. EVs harvested from MSCs and lipopolysaccharide (LPS) were used to stimulate the M064 cells to establish an in vitro inflammation cell model. Besides, 2,4,6‐trinitrobenzene sulfonic acid intracolon administration was performed to establish in vivo IBD mouse models. After loss‐ and gain‐of‐function assays, the regulatory role of MSCs‐derived EVs loaded with manipulated miR‐378a‐3p in IBD in relation to GATA2/AQP4/PPAR‐α were explored. Upregulation of GATA2 was identified in the colon tissue of IBD patients. GATA2, which was a target gene of miR‐378a‐3p, transcriptionally upregulated AQP4. After silencing of GATA2, LPS‐induced apoptosis of M064 cells was reduced by the downregulation of AQP4. Decreased AQP4 contributed to PPAR‐α pathway inactivation and weakened the LPS‐induced apoptosis of M064 cells. MSCs‐EVs delivering miR‐378a‐3p suppressed the GATA2/AQP4/PPAR‐α pathway, which reduced LPS‐induced apoptosis of M064 cells and the occurrence of IBD in mice. Altogether, the current study illustrated that MSCs‐EVs transfer miR‐378a‐3p to reduce the GATA2 expression, which downregulates AQP4 to block the PPAR‐α signalling pathway, thus suppressing the occurrence of IBD.

## 1 INTRODUCTION

Inflammatory bowel disease (IBD), classified as Crohn's disease and ulcerative colitis, is considered as chronic remittent/progressive inflammatory response affecting the entire gastrointestinal tract or the colonic mucosa.^1^ Pathologically, IBD is characterized by intestinal inflammation as well as extraintestinal manifestations such as keratopathy and episcleritis.^2^ As IBD is a multifactorial disorder, it is associated with various modification ranging from dietary, genetic, immunological and microbial factors.^3^ Statistically, the incidence of IBD is relatively low in Asian population relative to the Western population; however, a gradual rise has been apparent.^4^ Currently, surgical intervention is regarded as the gold standard for the treatment of IBD,^5^ and bacteriotherapy has been proposed as a promising protocol for the treatment of IBD.^6^

Mesenchymal stem cells (MSCs) have been identified as multipotent cells that are capable of proliferating or replacing dead cells in the body. MSCs also secrete immunomodulatory molecules.^7^ Recently, the application of MSCs has been proposed as an effective treatment protocol of IBD.^8^ Extracellular vesicles (EVs), including small EVs or exosomes, are small vesicles secreted by various cell types that can transfer intricate bioactive molecules for involvement in various pathophysiological pathways of intercellular communication.^9^ In light of existing research, microRNAs (miRs) can essentially function as efficient diagnostic molecules for the detection and classification in IBD subtypes.^10^ Intriguingly, miR‐378a‐3p could increase the IL‐33 expression upon inflammation, which can exacerbate the pathogenesis of ulcerative colitis.^11^ Based on our bioinformatic analysis, GATA‐binding protein 2 (GATA2) was targeted by miR‐378a‐3p. An existing study identified GATA2 as a multi‐catalytic transcription gene, which could serve as a regulator of inflammatory processes.^12^ As previously reported, GATA2 can radically influence the inflammation in colitis.^13^ Aquaporin 4 (AQP4) has been identified as a type of transmembrane protein of the aquaporin family and a vital water channel in the mammalian brain.^14^ Moreover, an existing study determined that the lack of AQP4 could facilitate the alleviation of experimental colitis in a mouse model.^15^ Peroxisome proliferator‐activated receptors (PPARs) have been identified as ligand‐activated transcription genes with notable functionality as mediators of various biological processes in IBD.^16^ Strikingly, inhibited PPAR‐α action due to palmitoylethanolamide could facilitate the alleviation of ulcerative colitis.^17^ In light of the aforementioned literature, the current study sought to investigate whether MSCs‐derived EVs (MSCs‐EVs) containing miR‐378a‐3p could influence the development of IBD, with involvement of the GATA2/AQP4/PPAR‐α axis.

## 2 MATERIALS AND METHODS

### 2.1 Ethical approval

The present study was performed with approval of the ethics committee of the General Surgery Institute of Yangzhou, Northern Jiangsu People's Hospital, Clinical Medical College, Yangzhou University. All participators provided signed informed consent prior to enrolment. All animal experiments were in compliance with the recommendations for the care and use of laboratory animals issued by the *National Institutes of Health*.

### 2.2 Bioinformatics analysis

Through the Gene Expression Omnibus (GEO) database, we searched the IBD‐related expression dataset GSE35609 was searched from the GEO database, which comprises of 4 normal samples and 5 disease samples. With the normal samples serving as control, the R language ‘limma’ package was used for a comprehensive differential analysis. With |logFC| > 1 and *p* value <0.05 as the screening criteria, the differentially expressed genes (DEGs) were identified. The binding domain of GATA2 in the AQP4 promoter region was predicted using the Jaspar database, while the AQP4‐related genes were predicted by GeneMANIA database, and the related genes were enriched by KEGG pathway and analysed by the KOBAS3.0 database. A combination of the starBase database and DIANA database were used to predict the upstream miRNA of GATA2. The EVmiRNA database was used to identify the miRNAs in the EVs of MSCs, and the miRNAs with more than 400 expressions were selected for subsequent analysis.

### 2.3 Clinical tissue sample collection

Normal and inflamed colonic mucosa biopsy tissues were harvested from healthy controls (HC; *n* = 17) and active ulcerative colitis patients (IBD, *n* = 35), respectively, who underwent examination in the General Surgery Institute of Yangzhou, Northern Jiangsu People's Hospital, Clinical Medical College, Yangzhou University from a period between September 2016 to May 2019. The clinical characteristics of these patients are shown in Table S1. These tissue samples were analysed using a combination of reverse transcription‐quantitative polymerase chain reaction (RT‐qPCR) and immunohistochemistry.

### 2.4 Isolation and culture of mouse mesenchymal stem cells

Bone marrow cells were rinsed from the mouse femurs and tibias with phosphate buffered saline (PBS) solution containing 2% foetal bovine serum (FBS, BI). Next, the cells were seeded (1 × 10^7^ cells per dish) in a 100‐mm dish (Corning Glass Works) for incubation in a 37°C incubator containing 5% CO_2_. After 2 days of culture, the adherent cells were cultured in α‐MEM (Invitrogen) supplemented with a combination of 20% FBS, 2 mM L‐glutamine (Invitrogen), 55 μm 2‐mercaptoethanol (Invitrogen), 100 μg/ml streptomycin and 100 U/ml penicillin (Invitrogen) for 14 days. Upon attaining 80%–90% confluence, the cells were subcultured for subsequent experimentation.

### 2.5 Isolation and identification of extracellular vesicles

The MSCs were cultured in complete medium containing FBS (centrifuged at 100,000× *g* for 2 h at 4°C to remove EVs). After incubation of the MSCs at the second passage for 48–72 h, the supernatant of MSCs was isolated and the EVs were extracted from MSCs (1.2 × 10^7^ cells) by differential centrifugation (centrifuged at 300× *g* for 10 min, 3000× *g* for 10 min, and 20,000× *g* for 30 min). The number of EVs was quantified using the EXOCEP EVs Quantification Kit (System Biosciences Inc.).

Next, MSCs‐EVs were subject to comprehensive evaluation. Briefly, 20 μl EVs were dripped onto the copper wire, and the liquid was dried from the side using filter paper after allowing to rest for 3 min. Subsequently, 30 μl of the phosphotungstic acid solution (pH 6.8) was dripped onto the wire and re‐dyed at room temperature for 5 min. Next, the MSC‐EVs observations were documented under transmission electron microscopy (TEM). The particle size analysis^18^ was performed using a nanoparticle tracking analyzer (NTA; NS300, Malvern Instruments Ltd.). Western blot assay was performed using anti‐CD63 (sc‐5275) and anti‐CD9 (sc‐9148), and calnexin (sc‐23954).

### 2.6 Extracellular vesicles uptake assay

The purified MSCs‐EVs were incubated with PKH26 (Sigma‐Aldrich Chemical Company) for 5 min at ambient temperature. The labelled MSCs‐EVs were resuspended in basal medium prior to incubation with M064 cells at 37°C for 12 h. Next, 4′6‐diamidino‐2‐phenylindole (DAPI; Sigma‐Aldrich Chemical Company) was used to stain the nuclei for 10 min. The stained cells were observed under the IX53 fluorescence microscope (Olympus).

### 2.7 Cell culture and establishment of inflammation model in vitro

Mouse colonic epithelial cell line M064 (SNPM‐M064; Sunncell) and H&EK 293T cells (ATCC) were cultured in Dulbecco's modified Eagle medium (DMEM) (Gibco) medium containing a combination of 10% FBS, 100 μg/ml streptomycin and 100 U/ml penicillin in a 37°C incubator containing 5% CO_2_. Upon attaining 80%–90% cell confluence, the cells were passaged. The M064 cells were reacted with 1 μg/ml LPS (Sigma‐Aldrich Chemical Company, prepared with 0.1% DMSO) for 12 h to successfully establish an in vitro inflammation model.^19^ Meanwhile, the control cells were treated with 0.1% DMSO.

### 2.8 Cell transfection and grouping

In strict accordance with the provided GATA2 and AQP4 sequences in NCBI, Sangon (Shanghai, China) was entrusted to construct the following plasmids: si‐GATA2, oe‐GATA2, si‐NC, oe‐NC, oe‐AQP4, si‐AQP4, as well as mimic NC, miR‐378a‐3p mimic, agomir NC and miR‐378a‐3p agomir.

The M064 cells were trypsinized and seeded into a 24‐well plate for growth in a monolayer. The culture medium was discarded, and the cells were transfected using Lipofectamine 2000 (11668–019, Invitrogen) as follows: (1) si‐NC and si‐GATA2; (2) si‐NC + oe‐NC, si‐GATA2 + oe‐NC and si‐GATA2 + oe‐AQP4; (3) si‐NC and si‐AQP4; (4) si‐NC + DMSO, si‐AQP4 + DMSO and si‐AQP4 + fenofibrate, a PPAR‐α agonist (50 μm, 24 h of reaction; APExBIO). The concentration of si‐NC, si‐GATA2 and si‐AQP4 was 100 nm, while that of oe‐NC, oe‐GATA2 and oe‐AQP4 was 50 nm. After 6–8 h of culture at 37°C with 5% CO_2_, the complete medium was renewed. After 48 h of culture, the RNA and protein contents were extracted for subsequent experimentation.

The miR‐378a‐3p mimic was transfected into MSCs according to the provided instructions, and the riboFECT™CP reagent (RiboBio) was added to transfect miR‐378a‐3p mimic or mimic NC (20 nmol) into 2 × 10^5^ MSCs. After 48 h of culture, MSCs‐EVs containing miR‐378a‐3p and MSCs‐EVs‐mimic NC were harvested through isolation and immediately co‐cultured with the M064 cells for subsequent detection.

### 2.9 Cell counting kit‐8 (CCK‐8) assay

M064 cells were seeded in a 96‐well plate at the density of 10^4^ cells per well for incubation in an incubator for 24 h. After LPS treatment for 12 h, 10 μl of the CCK‐8 (Sigma‐Aldrich Chemical Company) solution was added to each well. After incubation in a humidified incubator at 37℃ for 1 h, the absorbance value of each sample was estimated at the excitation wavelength of 450 nm with an Epoch microplate spectrophotometer (Bio‐Tek). Six duplicated wells were set.

### 2.10 Dual‐luciferase reporter gene assay

GATA2 3'‐UTR sequence containing the predicted miR‐378a‐3p binding site was inserted into pGL3 basic vector (Promega) of the XbaI restriction site downstream of luciferase gene to synthesise the firefly/Renilla luciferase reporter vector pGL3‐basic‐GATA2‐3'‐UTR‐wide type (WT) (GATA2‐WT). The mutant type (MUT) was pGL3‐basic‐GATA2‐3'‐UTR‐MUT (GATA2‐MUT; sequence: F: GGGGAGCTCTTCAAACAGGGAGATAATTTTAA; R: CCCCTCGAGACTCCTGCACAGACGTGAAG).^20^ H&EK‐293T cells were seeded in a 24‐well plate and incubated for 24 h. Upon attaining 50%–60% cell confluence, the cells were co‐transfected using Lipofectamine 2000 with NC‐mimic/miR‐378a‐3p mimic and GATA2‐WT; NC‐mimic/miR‐378a‐3p mimic and GATA2‐MUT. In addition, all cells were transfected with 10 ng of the pRL‐TK Renilla luciferase for 24 h. The luciferase activity was determined in the cell lysate. The relative luciferase activity was determined using the dual‐luciferase reporter gene assay system (E1910; Promega) and normalized to Renilla luciferase activity.

### 2.11 Chromatin immunoprecipitation (ChIP)

Cells were fixed using 1% formaldehyde for 10 min and terminated with the addition of 0.125 m glycine. The cells were scraped off, and the cell precipitates were isolated by centrifugation. The cells were resuspended in cell lysis buffer (20 mm Tris HCl, pH 8.0, 85 mM KCl, 0.5% NP40 and protease inhibitor) and then centrifuged to isolate the nuclei. Nuclear precipitates were lysed in sodium dodecyl sulphate (SDS) lysis buffer (1% SDS, 10 mm ethylene diamine tetraacetic acid (EDTA), 50 mm Tris HCl, pH 8.1 and protease inhibitor) and then ultrasonically treated to fragment the DNA into segments of length between 200 and 1000 bp. The diluted ultrasonic lysate was added for ChIP, followed by incubation with the anti‐GATA2 (SC‐267, Santa Cruz Biotechnology) and anti‐IgG antibodies at 4℃ overnight respectively. Using the pierce protein A/G magnetic beads (88803, Thermo Fisher Scientific), GATA2 DNA could bind by centrifugation at 12,000× *g* for 5 min. Non‐specific complex was rinsed from the precipitates and crosslinked overnight at 65°C. The recovered and purified DNA fragments were subsequently used as the amplification templates. The primer sequence of AQP4 promoter was as follows: F: 5'‐GTGAGGAAATGCAGTGCCAA‐3', R: 5'‐CCCCATAGCAAACTAAGGGCT‐3'.^21^ The immunoprecipitated DNA was detected by qRT‐PCR using iQ SYBR Green supermax (Bio‐Rad Laboratories).

### 2.12 Coimmunoprecipitation (Co‐IP)

M064 cells were lysed in the IP lysis buffer (P0013; Beyotime Biotechnology) according to the provided instructions. Cell lysate containing 200 μg of protein was incubated with the Dynabeads^®^ Protein G and 2 μg AQP4 antibody (at a dilution ratio of 1: 100, sc‐32739, Santa Cruz Biotechnology) or IgG (negative control) at 4°C for 4 h. Finally, the immunoprecipitation complex was analysed by Western blot using the PPAR‐α antibody (at a dilution ratio of 1:100, SC‐398394, Santa Cruz Biotechnology).

### 2.13 qRT‐PCR

The Trizol reagent (15596026, Invitrogen) was used to extract the total RNA content from colon tissues and cells. The RNA content was reversely transcribed into cDNA using the PrimeScript ^RT^ reagent kit (RR047A, Takara, Otsu). The Poly A tailed detection kit (b532451, Sangon; including Universal PCR primer R and U6 Universal PCR primer R) was adopted to isolate the cDNA content from the miRNA containing Polya tail. Fast SYBR Green PCR kit (Applied Biosystems) and ABI prism 7300 qRT‐PCR system (Applied Biosystems) were used for qRT‐PCR detection. U6 served as the internal reference for miR‐378a‐3p, and glyceraldehyde‐phosphate dehydrogenase (GAPDH) served as internal reference for GATA2 and AQP4. The relative gene expression was analysed based on the 2^−ΔΔCT^ method. The primer design is provided in Table S2.

### 2.14 Western blot assay

The tissues and cells were isolated for lysis with the radioimmunoprecipitation assay (RIPA) lysate (Beyotime) containing 1% phenylmethylsulfonyl fluoride (PMSF) for 30 min on ice. The protein concentration was determined based on the BCA (Pierce) method. The protein content was denatured using 5× loading buffer and boiled at 10°C for 10 min; the loading amount of the protein was 50 μg. The separation gel and concentration gel were prepared for electrophoresis. After electrophoresis, the bands containing the target protein were transferred onto a polyvinylidene fluoride membrane. Next, the membrane was immersed in 5% skimmed milk powder, sealed at ambient temperature for 1 h and treated with mouse anti‐GATA2 (at a dilution ratio of 1:200, sc‐267, Santa Cruz Biotechnology), mouse anti‐AQP4 (at a dilution ratio of 1:200, sc‐32739, Santa Cruz Biotechnology), PPAR‐α (at a dilution ratio of 1:200, sc‐398394, Santa Cruz Biotechnology), mouse anti‐AQP4 (at a dilution ratio of 1:200, sc‐32739, Santa Cruz Biotechnology), PPAR‐α (at a dilution ratio of 1:200, sc‐398394, Santa Cruz Biotechnology), mouse Anti‐β‐actin (at a dilution ratio of 1:10,000, AC004, Abclonal Technology) overnight at 4°C. β‐actin served as an internal reference. Horseradish peroxidase (HRP)‐labelled goat anti‐mouse or goat anti‐rabbit against IgG (at a dilution ratio of 1:10,000, Boster) secondary antibody was added for incubation of the protein bands for 1 h at ambient temperature. The Electrogenerated chemiluminescence (ECL) reaction solution (Thermo Fisher Scientific) was evenly added to the membrane, which was then placed in the development instrument (Amersham Imager 600, Amersham Biosciences) for exposure. The Image J (NIH) software was used for grey value analysis.

### 2.15 Flow cytometry

Annexin v‐fluorescein isothiocyanate (FITC)/propidium iodide (PI) double staining kits (Article No.: 70‐AP101‐100, Lianke) were used to detect the degree of cell apoptosis. The cultured cells were digested with 0.25% trypsin (without EDTA) and centrifuged at 300× g for at least 5 min. After removal of the supernatant, the remaining precipitates were resuspended in 500 μl of binding buffer. Next, the samples were incubated with 3 μl of annexin V‐FITC and 2.5 μl of PI in conditions devoid of light at 37°C for 20 min. A flow cytometer (FACS, Calibur, BD Biosciences) was used to analyse the apoptosis of cells, and the observations were documented and analysed.

### 2.16 Establishment and of inflammatory bowel disease mouse model

A total of 50 male C57BL/6 mice (6 weeks old, weighing 18–22 g) were purchased from Shanghai SLAC Laboratory Animal Co., Ltd. The mice were housed at saturated humidity (45%–50%) and temperature (25–27°C) for 1 week with daily 12‐h light/darkness cycles for acclimation to the experimental environment. The mice were fasted for 12 h before administration, and they had ad libitum access to food and water at other times. Next, 2,4,6‐trinitrobenzene sulfonic acid (TNBS) was administered to the mice to establish the IBD model.^22^ Briefly, the mice were anaesthetized by inhalation of diethyl ether (Sigma‐Aldrich Chemical Company). Then, a 3‐cm catheter was inserted into the anus. Next, 3.5 mg TNBS was dissolved in 100 μl of 30% ethanol solution and administered in the colon via the catheter. The control mice were given 100 μl of 30% ethanol solution under similar protocol. The IBD mice were used as control or treated with TNBS, with 10 mice in each group. The IBD model mice were treated with MSCs‐EVs agomir NC + oe‐NC, MSCs‐EVs‐miR‐378a‐3p agomir +oe‐NC or MSCs‐EVs‐miR‐378a‐3p agomir + oe‐GATA2, with 10 mice in each group. TNBS was administrated once a week. During the first and second regimens of TNBS administration, 200 μg of MSCs‐EVs agomir NC or MSCs‐EVs‐miR‐378a‐3p agomir and 50 nmol of oe‐NC or oe‐GATA2 were injected intravenously into the mice. After 4 regimens of TNBS administration, the mice were euthanized by CO_2_ asphyxiation, and the colitis tissues were isolated about 2 cm away from ileocecal region for follow‐up detection.

### 2.17 Determination of disease activity index (DAI)

Several parameters such as the body weight, faecal characteristics and faecal occult blood were documented. DAI was calculated according to the scoring system provided in Table S3.

### 2.18 Haematoxylin and eosin staining

The colon segment (about 2–3 cm long) was excised, and the colon length from cecum to anus was measured. Next, the segment was fixed using 4% paraformaldehyde, paraffin‐embedded and sectioned (5 μm). The paraffin sections were then stained with Haematoxylin and eosin. First, the prepared sections were taken for routine de‐waxing and gradient alcohol dehydration, then stained with haematoxylin (Solarbio) for 2 min and separated with 1% hydrochloric acid ethanol for 10 s. The sections were subsequently stained with eosin solution for 1 min, dehydrated with gradient alcohol, cleared with xylene and finally sealed with neutral gum. The histological modifications were all observed under an optical microscope (XP‐330, Shanghai Bingyu Optical Instrument Co., Ltd.). The total injury score was determined by the following parameters: goblet cell depletion (presence = 1, absence = 0), crypt abscess (presence = 1, absence = 0), mucosal structural destruction (normal = 1, moderate = 2, extensive = 3), muscle thickening (normal = 1, moderate = 2, extensive = 3) and cell infiltration (normal = 1, moderate = 2, transmural = 3).

### 2.19 Immunohistochemistry

The colonic mucosa was fixed using 4% paraformaldehyde for 1 week. Next, the samples were embedded in paraffin and divided into 4 μm thick sections. The sections were treated with 0.1 M citric acid buffer (pH 6.0) and heated in a 100°C microwave oven for 10 min to facilitate antigen repair. The sections were then subject to overnight incubation with the corresponding primary antibodies anti‐GATA2 (at a dilution ratio of 1:100, SC‐267, Santa Cruz Biotechnology), anti‐AQP4 (at a dilution ratio of 1:100, sc‐32739, Santa Cruz Biotechnology) and anti‐PPAR‐α (at a dilution ratio of 1:100, sc‐398394, Santa Cruz Biotechnology) at 4°C. Next, the secondary antibody goat anti‐rabbit (ab6721, Abcam Inc.) in combination with HRP was subject to incubation at room temperature for 1 h. Immune response was generated using 0.05% diaminobenzidine (DAB) containing 0.01% hydrogen peroxide (H_2_O_2;_ Bioss). The slices were soaked in haematoxylin for 5 min, then in 1% hydrochloric acid alcohol for 4 s to revert to blue colour. The standard of protein‐positive cells was identified as the normal positive cells which were brownish yellow. The sum of integrated optical density was analysed using the Image‐Pro Plus 6.0 software (Media Cybrnetics).

### 2.20 Statistical analysis

All experimental data, expressed as mean ± standard deviation, were analysed using the SPSS 21.0 statistical software (IBM) and graphPad Prism 7.0. The cell experiments were conducted three times independently. Data between two groups were compared by the unpaired *t* test and those among multiple groups were compared by one‐way analysis of variance (ANOVA), followed by Tukey's post hoc tests. In all statistical references, a value of *p* < 0.05, *p* < 0.01, *p* < 0.001 or *p* < 0.0001 was demonstrative of a statistically significant difference.

## 3 RESULTS

### 3.1 GATA2 expression was elevated in colonic tissues of IBD patients and mice

**FIGURE 1:**
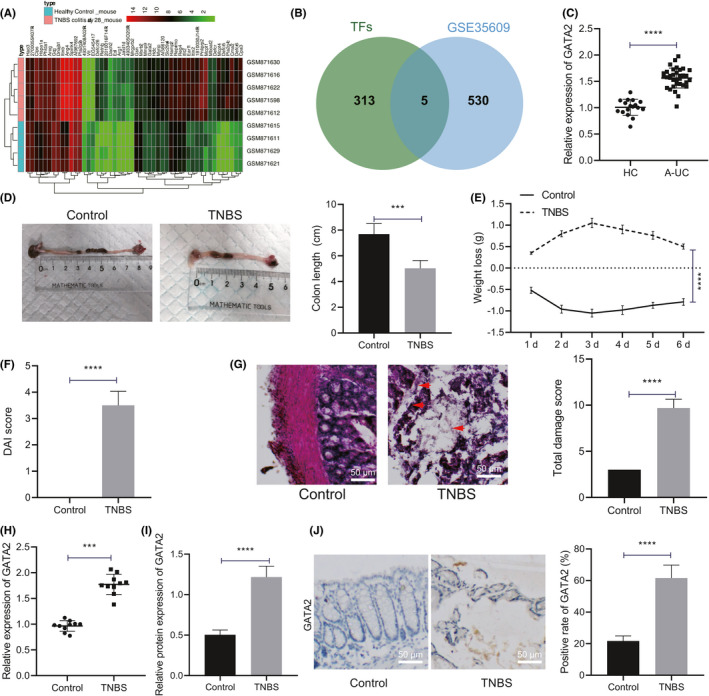
GATA2 is highly expressed in colonic tissues of IBD patients and mice. (A) The heatmap of differential gene expression pattern of IBD microarray was plotted. The sample number is shown in x‐axis, and gene name is shown in y‐axis; gene expression level clustering is shown in the dendrogram on the left, sample clustering in the dendrogram on the top and colour scale in the histogram on the right. (B) Retrieval of transcription factors in differential genes. Two circles in the figure represent transcription factors and differential genes, respectively, and the middle part represents the intersection of two groups of data. (C) qRT‐PCR was performed to detect GATA2 expression pattern in colonic mucosa of healthy controls (*n* = 17) and IBD patients (*n* = 35). (D) Colon length of mice with different treatment was recorded (*n* = 10). (E) Weight loss of mice with different treatment during administration was recorded (*n* = 10). (F) Statistical chart was plotted for DAI score of mice with different treatment (*n* = 10). (G) Haematoxylin and eosin staining was performed to determine colonic tissue damage of mice with different treatment (*n* = 10, scale bar: 50 μm) (H) qRT‐PCR was used to detect the mRNA expression pattern of GATA2 in colonic mucosa tissues of mice with different treatment (*n* = 10). (I) Western blot assay was used to detect the protein expression pattern of GATA2 in colonic mucosa tissues of mice with different treatment (*n* = 10). (J) The localization and expression pattern of GATA2 in mouse colonic mucosa tissues were determined with immunohistochemistry (*n* = 10, scale bar: 50 μm). Data between two groups were compared by unpaired *t* test and those among multiple groups at different timepoints by repeated measures of ANOVA, followed by Tukey's post hoc tests. ***p < 0.001. ****p < 0.0001

Initially, a differential analysis was performed on the expression microarray GSE35609 obtained from the GEO database, which identified 535 DEGs (Figure 1A). The 535 DEGs were further intersected with the transcription factors retrieved from Cistrome (Figure 1B), and five candidate transcription factors (GATA2, ATF3, BCl3, RXRG and ARNT) were determined. These five transcription factors showed significantly differential expression in IBD. Moreover, our findings revealed that GATA2 was the most DEG among the five candidate transcription factors (logFC = 1.8). Next, qRT‐PCR was conducted to detect the expression of GATA2 in the colonic mucosa of healthy controls and IBD patients. The results revealed that the expression pattern of GATA2 in the colonic mucosa of IBD patients was notably upregulated relative to the healthy controls (Figure 1C).

Subsequently, we established an IBD mouse model for further investigation. We initially detected alterations in the weight and colon length of mice (Figure 1D,E) and found that relative with the control mice, the weight of TNBS mice gradually decreased over time, and the colon length was also shortened significantly. Based on DAI score determination, relative to that in control mice, DAI of TNBS mice was markedly elevated (Figure 1F). Haematoxylin and eosin staining (Figure 1G) results elicited apparent inflammatory cell infiltration, crypt loss, mucosal destruction and oedema in the colon mucosa of TNBS mice, with an increased total injury fraction of TNBS mice in TNBS mice relative to the control mice. Subsequently, the results of qRT‐PCR and Western blot assay demonstrated a markedly increased expression pattern of GATA2 in TNBS mice relative to the control mice (Figure 1H,I, Figure S1A). Subsequently, immunohistochemistry was performed to determine the localization and expression pattern of GATA2 in the mouse colonic mucosa samples (Figure 1J), and the results displayed a predominant expression pattern of GATA2 in the nuclei of epithelial cells from the mouse colonic mucosa tissues, and its expression was markedly increased in TNBS mice in comparison with the control mice. The aforementioned results suggested that GATA2 was upregulated in the colonic mucosa of IBD patients and mice.

### 3.2 Silencing GATA2 promoted LPS‐induced proliferation and reduced the apoptosis of M064 cells by inhibiting AQP4 expression

**FIGURE 2:**
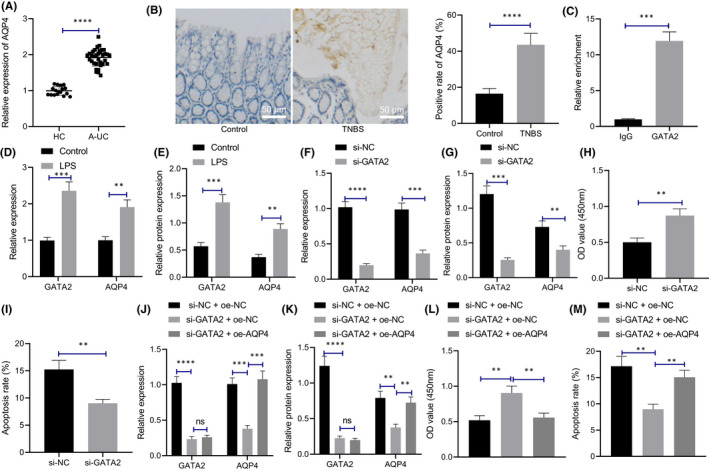
Silencing GATA2 reduces the apoptosis of M064 cells induced by LPS by inhibiting the expression pattern of AQP4. (A) qRT‐PCR was used to detect the expression pattern of AQP4 in colonic mucosa of healthy controls (*n* = 17) and IBD patients (*n* = 35). ***p < 0.001 vs. healthy controls. (B) The localization and expression pattern of AQP4 in mouse colonic mucosa tissues was determined with immunohistochemistry (*n* = 10, scale bar: 50 μm). (C) ChIP assay was used to detect the enrichment of GATA2 in AQP4 promoter region (***p < 0.001 vs. IgG antibody). (D) The mRNA expression levels of GATA2 and AQP4 in M064 cells stimulated by LPS were detected by qRT‐PCR (**p < 0.01 vs. control). (E) The protein expression levels of GATA2 and AQP4 in M064 cells stimulated by LPS were detected by Western blot assay. (F) The mRNA expression levels of GATA2 and AQP4 in M064 cells after silencing GATA2 were detected by qRT‐PCR. (G) The protein expression patterns of GATA2 and AQP4 in cells after silencing GATA2 were detected by Western blot assay. (H) Cell proliferation after silencing GATA2. (I) Cell apoptosis after silencing GATA2. (J) mRNA expression of GATA2 and AQP4 in cells with different treatment as determined by qRT‐PCR. (K) The protein expression patterns of GATA2 and AQP4 with different treatment were detected by Western blot assay. (L) CCK‐8 assay was performed to determine cell proliferation. (M) Flow cytometry was performed to detect cell apoptosis. Data between two groups were compared by unpaired *t* test and those among multiple groups by one‐way ANOVA, followed by Tukey's post hoc tests. **p < 0.01. ***p < 0.001. ****p < 0.0001. Cell experiments were conducted three times independently

We initially detected the expression pattern of AQP4 in the colonic mucosa of healthy controls and patients with IBD (Figure 2A) and determined that relative to the healthy controls, the AQP4 expression in the colonic mucosa of patients with IBD was significantly upregulated. The results of immunohistochemistry (Figure 2B) elicited a prominent expression pattern of AQP4 in the cell membrane of epithelial cells from the mouse colonic mucosa tissues, and its expression pattern was markedly elevated in the TNBS mice relative to the control mice. Therefore, we speculate that GATA2 may elevate the expression pattern of AQP4 through transcription, thus increasing the occurrence of IBD.

Subsequently, we investigated the binding condition of GATA2 to the AQP4 promoter region, and identified multiple GATA2 binding domains in the AQP4 promoter region (Table S4). Subsequent verification by ChIP revealed notable enrichment of GATA2 in the promoter region of AQP4 (Figure 2C), thereby eliciting the ability of GATA2 to bind to the promoter region of AQP4. Therefore, LPS was used to stimulate the M064 cells to establish an in vitro inflammation model to explore the regulatory effect of GATA2 on AQP4 expression pattern. LPS treatment increased the expression patterns of GATA2 and AQP4 in the M064 cells relative to the control cells (Figure 2D,E, Figure S1B). Furthermore, qRT‐PCR and Western blot assay (Figure 2F,G, Figure S1C) revealed evidently reduced GATA2 and AQP4 levels in M064 cells upon treatment with siRNA (si)‐GATA2 relative to the M064 cells with si‐NC treatment, hence suggesting that GATA2 could combine with the promoter region of AQP4 to promote the AQP4 expression pattern. CCK‐8 assay and flow cytometry (Figure 2H,I, Figure S2A) demonstrated that GATA2 silencing promoted the M064 cell proliferative ability and inhibited LPS‐induced M064 cell apoptotic capability.

To further explore whether silencing GATA2 can reduce the apoptosis of M064 cells induced by LPS by regulating the expression pattern of AQP4, we subsequently silenced GATA2 and overexpressed AQP4 in the M064 cells. qRT‐PCR and Western blot assay (Figure 2J,K, Figure S1D) revealed that si‐GATA2 treatment individually decreased GATA2 and AQP4 levels in LPS‐treated M064 cells, while further overexpression of AQP4 led to a marked increase in AQP4 expression pattern. Subsequently, CCK‐8 assay and flow cytometry (Figure 2L,M, Figure S2B) revealed that the silencing of GATA2 increased proliferation and decreased apoptosis of LPS‐treated M064 cells, which was further annulled by additional overexpression of AQP4. Overall, our findings elicited that silencing GATA2 can definitively reduce the LPS‐induced apoptosis of M064 cells by decreasing the expression of AQP4.

### 3.3 Silencing AQP4 inhibited PPAR‐α pathway to promote LPS‐induced proliferation and reduce apoptosis of M064 cells

**FIGURE 3:**
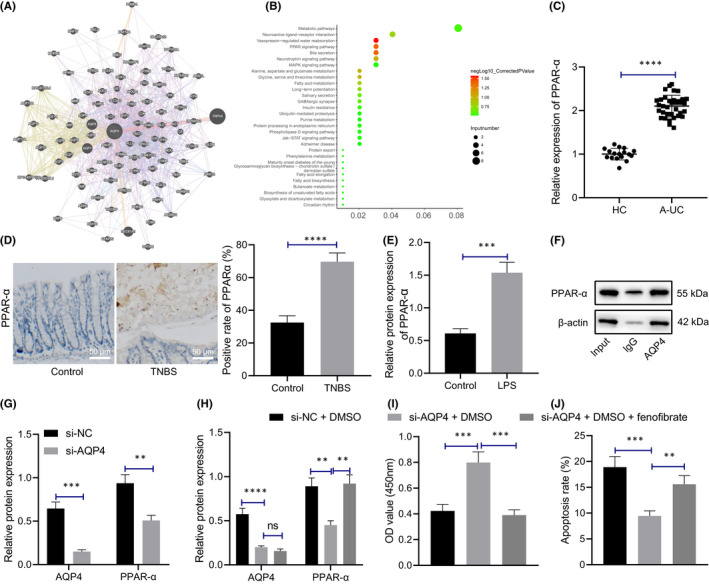
Silencing AQP4 inhibits PPAR‐α pathway and reduces LPS‐induced apoptosis of M064 cells. (A) Search of AQP4‐related genes. Each circle in the figure represents a gene, and the lines between circles indicate that there is correlation between genes. (B) KEGG pathway enrichment analysis of AQP4‐related genes. X‐axis represents GeneRatio, y‐axis represents functional items, circle size represents the number of enriched genes in the items, colour represents enrichment *p* value, and the right histogram is colour scale. (C) qRT‐PCR was used to detect the expression of PPAR‐α in colonic mucosa of healthy controls (*n* = 17) and patients with IBD (*n* = 35). (D) The localization and expression of PPAR‐α in mouse colonic mucosa tissues were determined with immunohistochemistry (*n* = 10, scale bar: 50 μm). (E) Western blot assay was used to detect the protein expression pattern of PPAR‐α in M064 cells after LPS stimulation. (F) The interaction between AQP4 and PPAR‐α in M064 cells was determined by Co‐IP, and its expression was subjected to Western blot assay. (G) Western blot analysis was used to detect the protein expression patterns of AQP4 and PPAR‐α in M064 cells after silencing AQP4. (H) The protein expression patterns of AQP4 and PPAR‐α in M064 cells with different treatment were detected by Western blot analysis. (I) CCK‐8 assay was performed to detect cell proliferation. (J) Flow cytometry was performed to detect cell apoptosis. Data between two groups were compared by unpaired *t* test and those among multiple groups by one‐way ANOVA, followed by Tukey's post hoc tests. **p < 0.01. ***p < 0.001. ****p < 0.0001. Cell experiments were conducted three times independently

We searched the AQP4‐related genes (Figure 3A), performed KEGG pathway enrichment analysis, (Figure 3B) and found that the AQP4‐related genes were prominently enriched in the PPAR signalling pathway. PPAR‐α is a member of the nuclear receptor superfamily PPARs^21^, ^22^ notably expressed in the intestinal cells, and can serve as modulators of inflammation and immune response.^23^, ^24^, ^25^

We initially detected the expression pattern of PPAR‐α in the colonic mucosa of healthy controls and patients with IBD, which revealed a considerably elevated PPAR‐α expression in colonic mucosa of patients with IBD compared to the healthy controls (Figure 3C). Immunohistochemistry (Figure 3D) elicited identification of PPAR‐α in the nuclei of epithelial cells from the mouse colonic mucosa tissues, with a radically rising level in the TNBS mice compared to the control mice. Further detection of the PPAR‐α expression pattern in M064 cells after LPS treatment revealed that relative to the control cells, the PPAR‐α level was notably higher in LPS‐treated M064 cells (Figure 3E, Figure S1E).

Subsequently, we investigated the interaction between AQP4 and PPAR‐α through Co‐IP and identified that AQP4 could definitively bind to PPAR‐α (Figure 3F). qRT‐PCR revealed that compared to si‐NC, si‐AQP4 decreased AQP4 and PPAR‐α expression pattern in LPS‐treated M064 cells (Figure 3G). Furthermore, the M064 cells were further treated with fenofibrate. Fenofibrate has extensive application in reducing the cholesterol level of patients at risk of cardiovascular disease. It can be used alone or in combination with statins for the treatment of hypercholesterolemia and hypertriglyceridemia.^26^ Western blot results showed that si‐AQP4 contributed to the significantly decreased protein expression pattern of AQP4 and PPAR‐α in the presence of DMSO, while additional treatment of fenofibrate notably increased the PPAR‐α expression pattern (Figure 3H). Moreover, M064 cell proliferation increased and apoptosis decreased after si‐AQP4 and DMSO treatment, which was further annulled by fenofibrate administration (Figure 3I,J). The aforementioned results suggest that silencing AQP4 is capable of inhibiting PPAR‐α pathway and reducing LPS‐induced apoptosis of M064 cells.

### 3.4 miR‐378a‐3p targeted and inhibited GATA2

**FIGURE 4:**
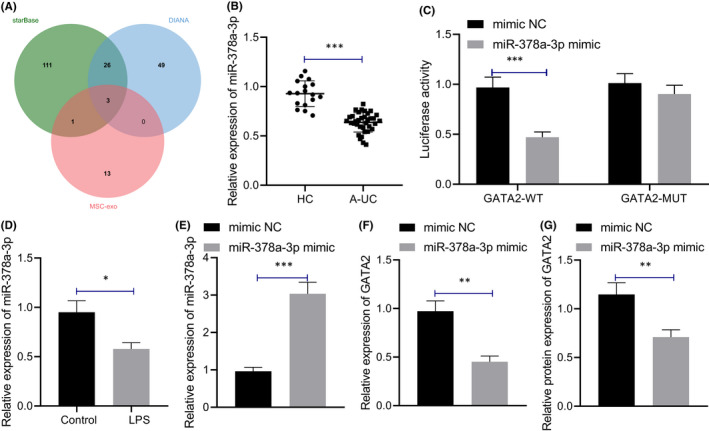
miR‐378a‐3p targets and inhibits GATA2. (A) Prediction of upstream miRs of GATA2. The three circles in the figure represent the prediction results of upstream miRs of GATA2 and the miR expressed in the MSCs‐EVs, and the middle part represents the intersection of three groups of data. (B) qRT‐PCR detection of miR‐378a‐3p expression pattern in colonic mucosa of healthy controls (*n* = 17) and patients with IBD (*n* = 35). (C) Dual‐luciferase reporter gene assay was used to detect the targeting relationship between miR‐378a‐3p and GATA2. (D) qRT‐PCR was used to detect the expression pattern of miR‐378a‐3p in M064 cells stimulated by LPS. (E) qRT‐PCR was used to detect the expression pattern of miR‐378a‐3p in M064 cells after overexpression of miR‐378a‐3p. (F) qRT‐PCR was used to detect the expression pattern of GATA2 in M064 cells after overexpression of miR‐378a‐3p. (G) The protein expression pattern of GATA2 in M064 cells after overexpression of miR‐378a‐3p was detected by Western blot assay. Data between two groups were compared by unpaired *t* test. *p < 0.05. **p < 0.01. ***p < 0.001. Cell experiments were conducted three times independently

We predicted the upstream miRs of GATA2 using scientific databases such as starBase and intersected the prediction results with the miRs expressed in the EVs of MSCs retrieved from EVmiRNA (Figure 4A). Finally, we identified three candidate miRs, namely miR‐25‐3p, miR‐92a‐3p and miR‐378a‐3p. Next, qRT‐PCR was performed to detect the expression pattern of miR‐378a‐3p in the colonic mucosa of healthy controls and patients with IBD, which revealed considerably reduced miR‐378a‐3p expression pattern in the colonic mucosa of patients with IBD relative to the healthy controls (Figure 4B). Intriguingly, the involvement of decreased miR‐378a‐3p has been identified in the pathogenesis of ulcerative colitis.^11^

Dual‐luciferase reporter gene assay displayed that compared with the mimic NC, miR‐378a‐3p mimic led to a notable reduction of luciferase activity in the GATA2‐wild‐type (WT) co‐transfection group (Figure 4C), thereby highlighting that miR‐378a‐3p can target GATA2. After LPS stimulation, a lowered miR‐378a‐3p expression pattern was evident in the M064 cells (Figure 4D). qRT‐PCR (Figure 4E,F) demonstrated that compared with mimic NC, miR‐378a‐3p mimic markedly upregulated the expression pattern of miR‐378a‐3p in LPS‐treated M064 cells, while that of GATA2 was significantly downregulated. Western blot assay revealed that the GATA2 protein level was decreased after miR‐378a‐3p elevation in LPS‐treated M064 cells (Figure 4G, Figure S1F). Conjointly, miR‐378a‐3p can target and inhibit GATA2 expression.

### 3.5 Mesenchymal stem cells‐derived extracellular vesicles carrying miR‐378a‐3p inhibited GATA2/AQP4/PPAR‐α pathway and reduced LPS‐induced apoptosis of M064 cells

**FIGURE 5:**
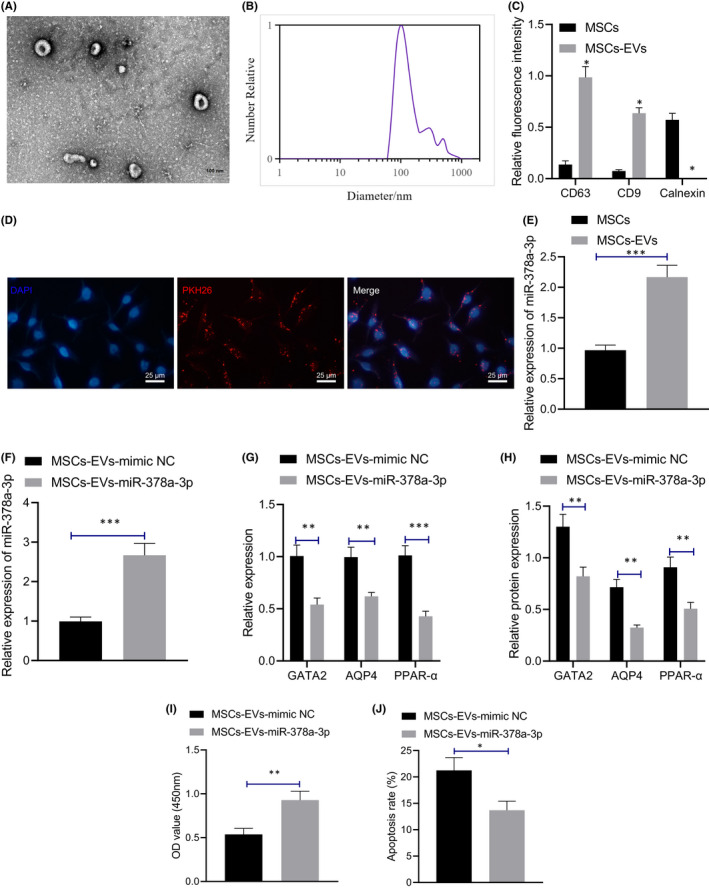
Mesenchymal stem cells‐derived extracellular vesicles carrying miR‐378a‐3p inhibits GATA2/AQP4/PPAR‐α pathway and reduced LPS‐induced apoptosis of M064 cells. (A) The vesicles in MSCs were analysed by transmission electron microscopy (500 nm). (B) The particle size of MSCs‐EVs was measured using a nanoparticle tracking analyzer. (C) The expression patterns of CD63, CD9 and calnexin in the EVs were detected by immunofluorescence and Western blot assay. (D) The uptake of MSCs‐EVs by M064 cells with different treatment was observed by fluorescence microscope (scale bar: 25 μm). (E) The expression pattern of miR‐378a‐3p in MSCs and MSCs‐EVs was detected by qRT‐PCR. (F) The expression pattern of miR‐378a‐3p in M064 cells with different treatment was detected by qRT‐PCR. (G) The mRNA expression patterns of GATA2 and AQP4 in M064 cells with different treatment were detected by qRT‐PCR. (H) The protein expression patterns of GATA2, AQP4 and PPAR‐α in M064 cells with different treatment were detected by Western blot assay. (I) The proliferation of M064 cells was detected by CCK‐8 assay. (J) The apoptosis of M064 cells was detected by flow cytometry. Data between two groups were compared by unpaired *t* test. *p < 0.05. **p < 0.01. ***p < 0.001. Cell experiments were conducted three times independently

In this experiment, we extracted EVs from MSCs by differential centrifugation for observation under transmission electron microscopy (TEM; Figure 5A), which revealed that the EVs were round‐ or cup‐shaped with a diameter of 100 nm. The particle size of purified MSCs‐EVs was measured using a nanoparticle tracking analyzer (NTA), demonstrating that the particle size of MSCs‐EVs ranged between 60 and 120 nm (Figure 5B). Immunofluorescence and Western blot results demonstrated that CD63 and CD9 were expressed in MSCs‐EVs, while calnexin was almost not expressed in MSCs‐EVs (Figure 5C, Figure S1G), thus indicative of the successful isolation of EVs from MSCs. As observed under fluorescence microscope (Figure 5D), the PKH26‐labelled MSCs‐EVs showed red fluorescence in the cytoplasm of M064 cells, indicating that MSCs‐EVs could be absorbed by the M064 cells. Moreover, our findings elicited a slightly higher miR‐378a‐3p expression pattern in MSCs‐EVs relative to the MSCs (Figure 5E).

We further transfected MSCs with the miR‐378a‐3p mimic or mimic NC, extracted MSCs‐EVs, and co‐cultured it with LPS‐induced M064 cells. qRT‐PCR results (Figure 5F,G) revealed that compared with the MSCs‐EVs‐mimic NC, MSCs‐EVs containing miR‐378a‐3p upregulated the miR‐378a‐3p expression pattern while downregulating the expression patterns of GATA2, AQP4 and PPAR‐α. Western blot assay (Figure 5H, Figure S1H) revealed that MSCs‐EVs‐miR‐378a‐3p markedly downregulated the GATA2, AQP4 and PPAR‐α protein levels in the LPS‐treated M064 cells in comparison with the MSCs‐EVs‐mimic NC. From CCK‐8 assay and flow cytometry (Figure 5I,J, Figure S2C), compared with the MSCs‐EVs‐mimic NC, MSCs‐EVs‐miR‐378a‐3p increased the proliferation of LPS‐treated M064 cells and reduced the apoptosis. Collectively, our results demonstrated that MSCs‐EVs carrying miR‐378a‐3p can repress the GATA2/AQP4/PPAR‐α pathway to impede LPS‐induced apoptosis of M064 cells.

### 3.6 Mesenchymal stem cells‐derived extracellular vesicles carrying miR‐378a‐3p inhibited GATA2/AQP4/PPAR‐α pathway and suppressed the occurrence of IBD in mice

**FIGURE 6:**
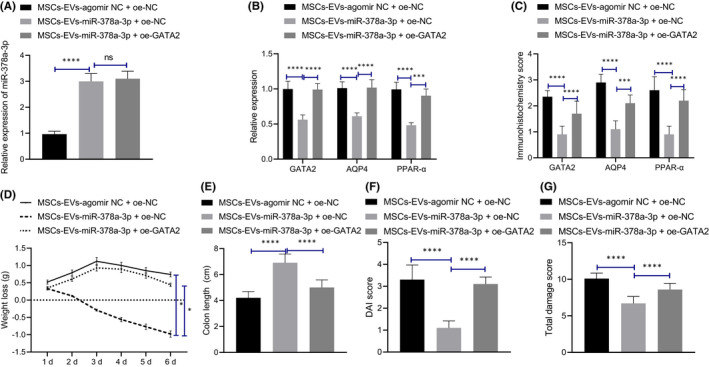
MSCs‐EVs carrying miR‐378a‐3p inhibits GATA2/AQP4/PPAR‐α pathway to suppress the occurrence of IBD in mice. (A) The expression pattern of miR‐378a‐3p in colonic mucosa tissues of mice with different treatment was detected by qRT‐PCR. (B) The mRNA expression patterns of GATA2, AQP4 and PPAR‐α in colonic mucosa tissues of mice with different treatment were detected by qRT‐PCR. (C) The expression patterns of GATA2, AQP4 and PPAR‐α in colonic mucosa tissues of mice with different treatment were detected by immunohistochemical staining. (D) Weight loss of mice with different treatment during administration was recorded. (E) Colon length of mice with different treatment was recorded. (F) The statistical plot was drawn for DAI score of mice with different treatment. (G) The statistical plot was plotted for colon tissue injury score of mice with different treatment as detected by Haematoxylin and eosin staining. Data among multiple groups were compared by one‐way ANOVA and those at different timepoints by repeated measures ANOVA, followed by Tukey's post hoc tests. *p < 0.05. ***p < 0.001. ****p < 0.0001. *n* = 10

We further explored whether MSCs‐EVs containing miR‐378a‐3p could regulate the GATA2/AQP4/PPAR‐α pathway to influence the occurrence of IBD in mice. Initially, qRT‐PCR (Figure 6A) showed that compared to the MSCs‐EVs‐agomir NC, MSCs‐EVs‐miR‐378a‐3p led to a notable increase in the expression pattern of miR‐378a‐3p in the colonic mucosa tissues of mice with the presence of overexpression (oe)‐NC, while no marked expression pattern alteration was evident in miR‐378a‐3p by subsequent treatment of oe‐GATA2. Furthermore, qRT‐PCR and Immunohistochemical results (Figure 6B,C) revealed that MSCs‐EVs‐miR‐378a‐3p markedly decreased GATA2, AQP4 and PPAR‐α expression pattern in the colonic mucosa tissues of mice, which was annulled by further treatment of oe‐GATA2.

Subsequently, we detected the weight and colon length modifications in mice with different treatment protocols (Figure 6D,E, Figure S3A), and determined that the weight of mice treated with MSCs‐EVs agomir NC +oe‐NC decreased gradually, while MSCs‐EVs‐miR‐378a‐3p increased the mouse weight and colon length; however, GATA2 elevation inverted the effect of MSCs‐EVs‐miR‐378a‐3p, resulting in marked weight loss with shortening of colon length. An analysis of the DAI score (Figure 6F) revealed that MSCs‐EVs‐miR‐378a‐3p resulted in significantly decreased DAI, while GATA2 elevation restored the protective effect of MSCs‐EVs‐miR‐378a‐3p. Based on the results from Haematoxylin and eosin staining (Figure 6G, Figure S3B), after treatment with MSCs‐EVs agomir NC, inflammatory cell infiltration, crypt loss, mucosal destruction and oedema were all evident in the colonic mucosa of mice, while MSCs‐EVs‐miR‐378a‐3p reduced the degree of damage of colonic mucosa, which was annulled by further GATA2 elevation. Altogether, MSCs‐EVs carrying miR‐378a‐3p could suppress the GATA2/AQP4/PPAR‐α pathway, thereby impeding the occurrence of IBD in mice.

## 4 DISCUSSION

Inflammatory bowel disease is regarded as a non‐communicable disease lacking effective pharmacological therapy.^27^ In the current study, we sought to determine the regulatory mechanism of miR‐378a‐3p‐containing MSCs‐EVs in IBD and identified that it could inhibit the progression of IBD by blockade of the AQP4/GATA2/PPAR‐α axis.

Initially, our findings elicited the elevation of GATA2 in the colon tissues of IBD patients and mice, and its silencing could reduce the LPS‐induced apoptosis of M064 cells. An existing study investigated the role of GATA2 in IBD and determined that the GATA2 deficiency might influence the development of IBD.^28^ Additionally, previous research identified that the transcription factor GATA2 serve as an inducer for the inflammatory reaction in colitis.^13^ Furthermore, the current study demonstrated that the inhibition of GATA2 could contribute to downregulated AQP4 and blockade of PPAR‐α, thereby reducing the LPS‐induced apoptosis of M064 cells. Notably, the interaction between GATA2 and AQP4 and that between AQP4 and PPAR‐α have been rarely reported. The results of the current study demonstrated a positive regulation in IBD in them. Besides, accumulating evidence has documented the participation of AQP4 and PPAR‐α in the development of IBD.^23^, ^29^ For instance, the involvement of AQP4 was identified in the pathogenesis of intestinal dysfunction after traumatic brain injury due to regulation of intestinal oedema.^30^ Suppression of PPAR‐α action by PEA could aid in the inhibition of ulcerative colitis.^17^ Strikingly, PPAR‐α activated by fenofibrate treatment could evidently exacerbate inflammation and tissue injury in acute colitis.^31^

Essentially, the current study also determined that MSCs‐EVs‐containing miR‐378a‐3p could reduce the LPS‐induced apoptosis of M064 cells and the occurrence of IBD in mice. An existing study has elicited the potential application of MSCs and EVs in the treatment of IBD. Previously, MSCs have demonstrated therapeutic potential in IBD.^32^ EVs elicit functionality in the aspect of immune mediation as well as intestinal barrier integrity and regulation of IBD.^33^ Besides, it was previously reported that exosomes derived from human umbilical cord MSCs could radically attenuate IBD in a mouse model.^34^ Moreover, EVs derived from human bone marrow‐derived MSCs could evidently alleviate colitis in IBD models and thus have been proposed as potential candidates in the treatment of IBD.^35^ Furthermore, several studies have identified the ability of EVs to deliver miRs to influence the progression of several diseases including those associated with IBD. For instance, EVs containing miR‐146a could target TRAF6 and IRAK1 to ameliorate experimental colitis.^36^ Moreover, miR‐200b‐containing microvesicles could evidently alleviate experimental colitis‐associated intestinal fibrosis via suppression of epithelial‐mesenchymal transition.^37^ The role of miR‐378a‐3p in intestinal diseases has been previously investigated. Existing findings revealed that miR‐378a‐3p elevated IL‐33 expression in an inflammatory environment, thereby inducing the pathogenesis of ulcerative colitis.^11^ Besides, CDK6 inhibition targeted by miR‐378a‐3p can serve as a protective barrier against intestinal injury induced by ionizing radiation.^38^ Mechanistically, our findings elicited that miR‐378a‐3p could target and inhibit the expression of GATA2. Therefore, after delivering M064 cells by MSCs‐EVs, miR‐378a‐3p downregulated GATA2 to block AQP4 and PPAR‐α, which radically inhibited IBD development.

**FIGURE 7:**
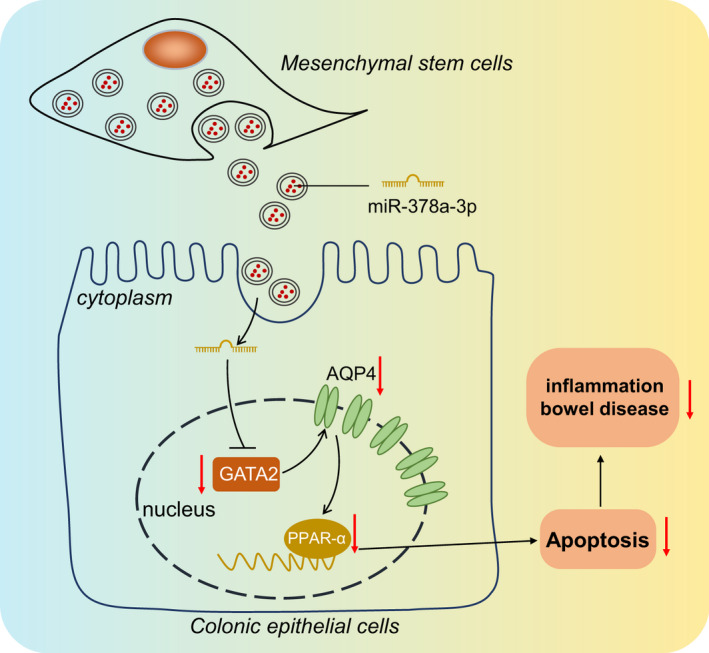
Molecular mechanism plot. MSCs‐EVs deliver miR‐378a‐3p to inhibit transcription factor GATA2, thus downregulating the expression pattern of AQP4 and blocking PPAR‐α signalling pathway, which inhibits the occurrence of IBD by suppression of LPS‐induced apoptosis of M064 cells

To conclude, the key findings in this study elicited that MSCs‐EVs could carry miR‐378a‐3p to inhibit the transcription factor GATA2, which can downregulate the expression of AQP4 and block PPAR‐α signalling pathway, thus inhibiting the occurrence of IBD (Figure 7). This study may provide a novel target and theoretical basis for the clinical treatment of IBD. However, further investigations are warranted to validate our results for clinical application.

## CONFLICT OF INTERESTS

The author declares no conflict of interest exists.

## AUTHOR CONTRIBUTIONS

**Ping Li:** Conceptualization (equal); Data curation (equal); Visualization (lead); Writing – original draft (lead). **Hai‐Yan Zhang:** Conceptualization (equal); Data curation (equal); Methodology (equal); Writing – original draft (equal). **Jian‐Zhen Gao:** Data curation (equal); Formal analysis (equal); Writing – review & editing (supporting). **Wen‐Qiang Du:** Investigation (equal); Software (lead); Writing – original draft (supporting). **Dong Tang:** Methodology (equal); Validation (equal); Writing – original draft (supporting). **Wei Wang:** Investigation (supporting); Validation (equal); Writing – review & editing (supporting). **Liu‐Hua Wang:** Formal analysis (equal); Project administration (lead); Writing – original draft (equal); Writing – review & editing (equal).

## Supporting information

Figure S1‐S3

Table S1‐S4

## Data Availability

The data and materials of the study can be obtained from the corresponding author upon request.

## References

[1] Kaser A , Zeissig S , Blumberg RS . Inflammatory bowel disease. Annu Rev Immunol. 2010;28:573‐621. 20192811 10.1146/annurev-immunol-030409-101225 PMC4620040

[2] Shah J , Shah A , Hassman L , Gutierrez A . Ocular manifestations of inflammatory bowel disease. Inflamm Bowel Dis. 2021;27:1832‐1838. 33501989 10.1093/ibd/izaa359

[3] Tarris G , de Rougemont A , Charkaoui M , Michiels C , Martin L , Belliot G . Enteric viruses and inflammatory bowel disease. Viruses. 2021;13:104. 33451106 10.3390/v13010104 PMC7828589

[4] Park J , Cheon JH . Incidence and prevalence of inflammatory bowel disease across Asia. Yonsei Med J. 2021;62:99‐108. 33527789 10.3349/ymj.2021.62.2.99 PMC7859683

[5] Crippa J , Carvello M , Kotze PG , Spinelli A . Robotic surgery in inflammatory bowel disease. Curr Drug Targets. 2021;22:112‐116. 33109059 10.2174/1389450121999200820125918

[6] Yoshimatsu Y , Mikami Y , Kanai T . Bacteriotherapy for inflammatory bowel disease. Inflamm Regen. 2021;41:3. 33441186 10.1186/s41232-020-00153-4 PMC7807454

[7] Vasanthan J , Gurusamy N , Rajasingh S , et al. Role of human mesenchymal stem cells in regenerative therapy. Cells. 2020;10:54. 10.3390/cells10010054 PMC7823630 33396426

[8] Ko JZ , Johnson S , Dave M . Efficacy and safety of mesenchymal stem/stromal cell therapy for inflammatory bowel diseases: an up‐to‐date systematic review. Biomolecules. 2021;11:82. 33440772 10.3390/biom11010082 PMC7827559

[9] Konkoth A , Saraswat R , Dubrou C , et al. Multifaceted role of extracellular vesicles in atherosclerosis. Atherosclerosis. 2021;319:121‐131. 33261815 10.1016/j.atherosclerosis.2020.11.006

[10] Alamdari‐Palangi V , Vahedi F , Shabaninejad Z , et al. microRNA in inflammatory bowel disease at a glance. Eur J Gastroenterol Hepatol. 2021;32:140‐148. 32558695 10.1097/MEG.0000000000001815

[11] Dubois‐Camacho K , Diaz‐Jimenez D , De la Fuente M , et al. Inhibition of miR‐378a‐3p by inflammation enhances IL‐33 levels: a novel mechanism of alarmin modulation in ulcerative colitis. Front Immunol. 2019;10:2449. 31824476 10.3389/fimmu.2019.02449 PMC6879552

[12] Muiya NP , Wakil S , Al‐Najai M , et al. A study of the role of GATA2 gene polymorphism in coronary artery disease risk traits. Gene. 2014;544:152‐158. 24786211 10.1016/j.gene.2014.04.064

[13] Kristensen NN , Olsen J , Gad M , Claesson MH . Genome‐wide expression profiling during protection from colitis by regulatory T cells. Inflamm Bowel Dis. 2008;14:75‐87. 17924563 10.1002/ibd.20277

[14] Kaptan S , Assentoft M , Schneider HP , et al. H95 Is a pH‐dependent gate in aquaporin 4. Structure. 2015;23:2309‐2318. 26585511 10.1016/j.str.2015.08.020

[15] Zhang Y , Zhang L , Wang Y , et al. KCNQ1OT1, HIF1A‐AS2 and APOA1‐AS are promising novel biomarkers for diagnosis of coronary artery disease. Clin Exp Pharmacol Physiol. 2019;46:635‐642. 30941792 10.1111/1440-1681.13094

[16] Mandard S , Patsouris D . Nuclear control of the inflammatory response in mammals by peroxisome proliferator‐activated receptors. PPAR Res. 2013;2013:613864. 23577023 10.1155/2013/613864 PMC3614066

[17] Esposito G , Capoccia E , Turco F , et al. Palmitoylethanolamide improves colon inflammation through an enteric glia/toll like receptor 4‐dependent PPAR‐alpha activation. Gut. 2014;63:1300‐1312. 24082036 10.1136/gutjnl-2013-305005

[18] Fang T , Lv H , Lv G , et al. Tumor‐derived exosomal miR‐1247‐3p induces cancer‐associated fibroblast activation to foster lung metastasis of liver cancer. Nat Commun. 2018;9:191. 29335551 10.1038/s41467-017-02583-0 PMC5768693

[19] Lin H , Honglang L , Weifeng L , Junmin C , Jiantao Y , Junjing G . The mechanism of alopolysaccharide protecting ulceralive colitis. Biomed Pharmacother. 2017;88:145‐150. 28103508 10.1016/j.biopha.2016.11.138

[20] Qin DD , Yang YF , Pu ZQ , et al. NR4A1 retards adipocyte differentiation or maturation via enhancing GATA2 and p53 expression. J Cell Mol Med. 2018;22:4709‐4720. 30044048 10.1111/jcmm.13715 PMC6156289

[21] Tome‐Garcia J , Erfani P , Nudelman G , et al. Analysis of chromatin accessibility uncovers TEAD1 as a regulator of migration in human glioblastoma. Nat Commun. 2018;9:4020. 30275445 10.1038/s41467-018-06258-2 PMC6167382

[22] Chen G , Ran X , Li B , et al. Sodium butyrate inhibits inflammation and maintains epithelium barrier integrity in a TNBS‐induced inflammatory bowel disease mice model. EBioMedicine. 2018;30:317‐325. 29627390 10.1016/j.ebiom.2018.03.030 PMC5952406

[23] Zhou X , Cao L , Jiang C , et al. PPARalpha‐UGT axis activation represses intestinal FXR‐FGF15 feedback signalling and exacerbates experimental colitis. Nat Commun. 2014;5:4573. 25183423 10.1038/ncomms5573 PMC4164778

[24] Gu X , Song Y , Chai Y , et al. GC‐MS metabolomics on PPARalpha‐dependent exacerbation of colitis. Mol Biosyst. 2015;11:1329‐1337. 25790429 10.1039/c5mb00048c PMC6334306

[25] Wang L , Xie H , Xu L , et al. rSj16 protects against DSS‐induced colitis by inhibiting the PPAR‐alpha signaling pathway. Theranostics. 2017;7:3446‐3460. 28912887 10.7150/thno.20359 PMC5596435

[26] Sidhu G , Tripp J . Fenofibrate. StatPearls; 2021. 32644645

[27] Di Stasi LC . Coumarin derivatives in inflammatory bowel disease. Molecules. 2021;26:422. 10.3390/molecules26020422 PMC7830946 33467396

[28] Amarnani AA , Poladian KR , Marciano BE , et al. A panoply of rheumatological manifestations in patients with GATA2 deficiency. Sci Rep. 2020;10:8305. 32433473 10.1038/s41598-020-64852-1 PMC7239896

[29] Vojdani A , Vojdani E , Herbert M , Kharrazian D . Correlation between antibodies to bacterial lipopolysaccharides and barrier proteins in sera positive for ASCA and ANCA. Int J Mol Sci. 2020;21:1381. 10.3390/ijms21041381 PMC7073094 32085663

[30] Duan H , Hao C , Fan Y , et al. The role of neuropeptide Y and aquaporin 4 in the pathogenesis of intestinal dysfunction caused by traumatic brain injury. J Surg Res. 2013;184:1006‐1012. 23622727 10.1016/j.jss.2013.03.096

[31] Qi Y , Jiang C , Tanaka N , et al. PPARalpha‐dependent exacerbation of experimental colitis by the hypolipidemic drug fenofibrate. Am J Physiol Gastrointest Liver Physiol. 2014;307:G564‐G573. 25035112 10.1152/ajpgi.00153.2014 PMC4154119

[32] Mao F , Tu Q , Wang L , et al. Mesenchymal stem cells and their therapeutic applications in inflammatory bowel disease. Oncotarget. 2017;8:38008‐38021. 28402942 10.18632/oncotarget.16682 PMC5514968

[33] Valter M , Verstockt S , Finalet Ferreiro JA , Cleynen I . Extracellular vesicles in inflammatory bowel disease: small particles, Big Players. J Crohns Colitis. 2021;15:499‐510. 32905585 10.1093/ecco-jcc/jjaa179

[34] Mao F , Wu Y , Tang X , et al. Exosomes derived from human umbilical cord mesenchymal stem cells relieve inflammatory bowel disease in mice. Biomed Res Int. 2017;2017:5356760. 28589143 10.1155/2017/5356760 PMC5447283

[35] Liu H , Liang Z , Wang F , et al. Exosomes from mesenchymal stromal cells reduce murine colonic inflammation via a macrophage‐dependent mechanism. JCI Insight. 2019;4:e131273. 10.1172/jci.insight.131273 PMC6975270 31689240

[36] Wu H , Fan H , Shou Z , et al. Extracellular vesicles containing miR‐146a attenuate experimental colitis by targeting TRAF6 and IRAK1. Int Immunopharmacol. 2019;68:204‐212. 30654310 10.1016/j.intimp.2018.12.043

[37] Yang J , Zhou CZ , Zhu R , et al. miR‐200b‐containing microvesicles attenuate experimental colitis associated intestinal fibrosis by inhibiting epithelial‐mesenchymal transition. J Gastroenterol Hepatol. 2017;32:1966‐1974. 28370348 10.1111/jgh.13797

[38] Chu X , Zheng W , Wang J , Zhang J , Pan Y , Shao C . CDK6 inhibition targeted by miR‐378a‐3p protects against intestinal injury induced by ionizing radiation. Biochem Biophys Res Commun. 2020;531:328‐334. 32800335 10.1016/j.bbrc.2020.07.093

