# Supplementary material for: Mesenchymal stem cells‐derived extracellular vesicles containing miR‐378a‐3p inhibit the occurrence of inflammatory bowel disease by targeting GATA2
Source: J Cell Mol Med. 2022 May 17;26(11):3133–46. doi: 10.1111/jcmm.17176 (PMC9170824; doi:10.1111/jcmm.17176)
Supplement: Supplementary file 1 — Figure S1‐S3 [file JCMM-26-3133-s002.docx]

**
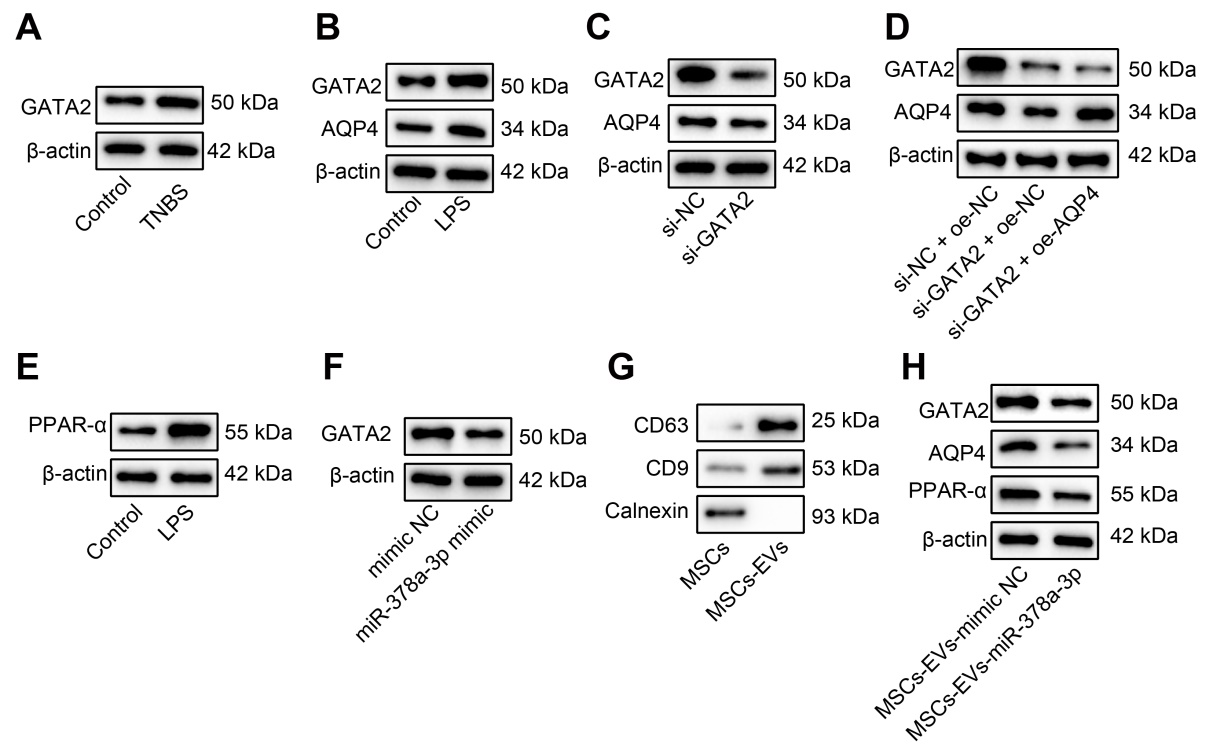
**

**FIGURE S1** A, Representative protein band of Figure 1I. B, Representative protein band of Figure 2E. C, Representative protein band of Figure 2G. D, Representative protein band of Figure 2K. E, Representative protein band of Figure 3E. F, Representative protein band of Figure 4G. G, Representative protein band of Figure 5C. H, Representative protein band of Figure 5H.

**
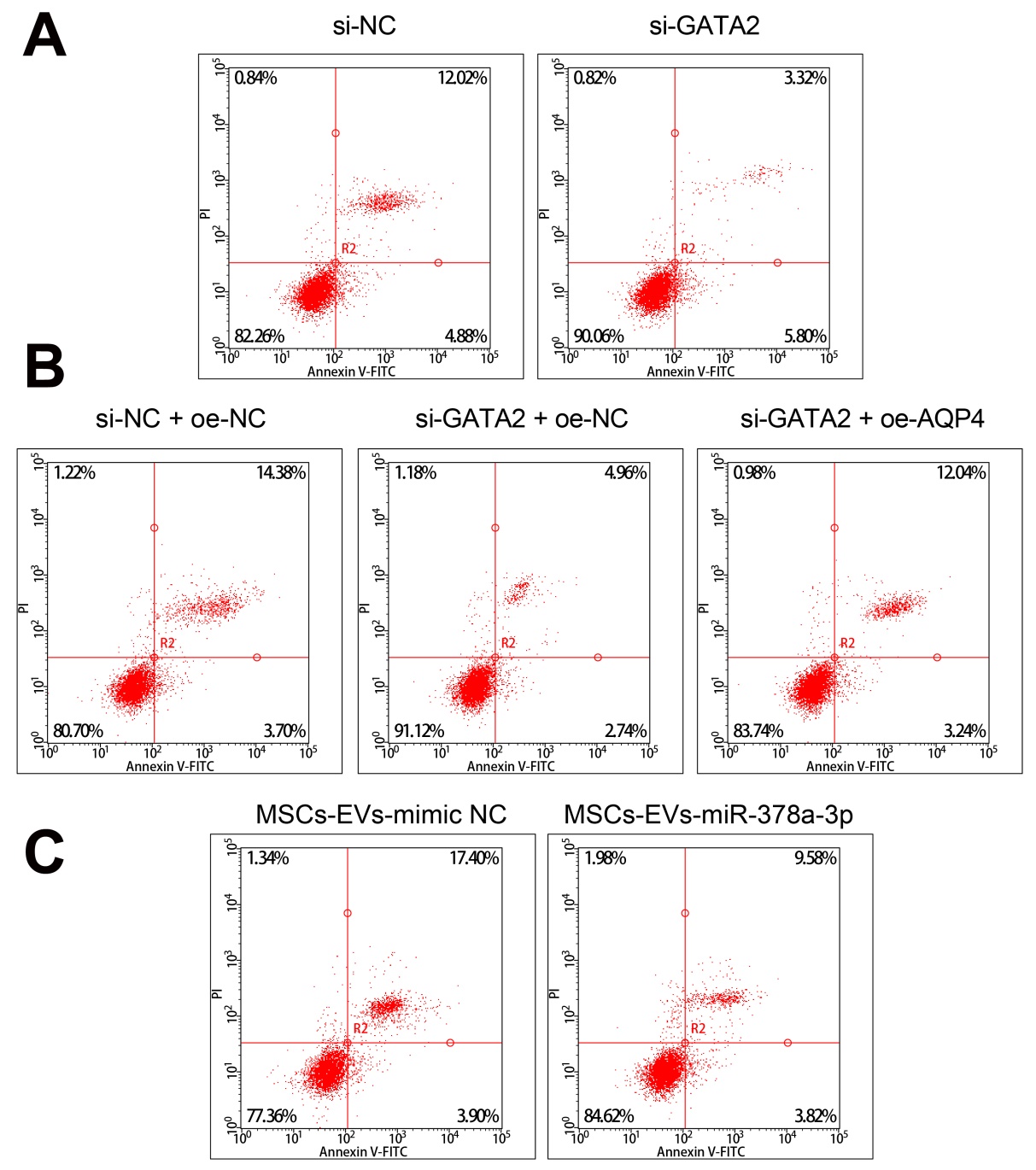
**

**FIGURE S2** A, Flow cytometry for Figure 2H. B, Flow cytometry for Figure 2L. C, Flow cytometry for Figure 5J.

**
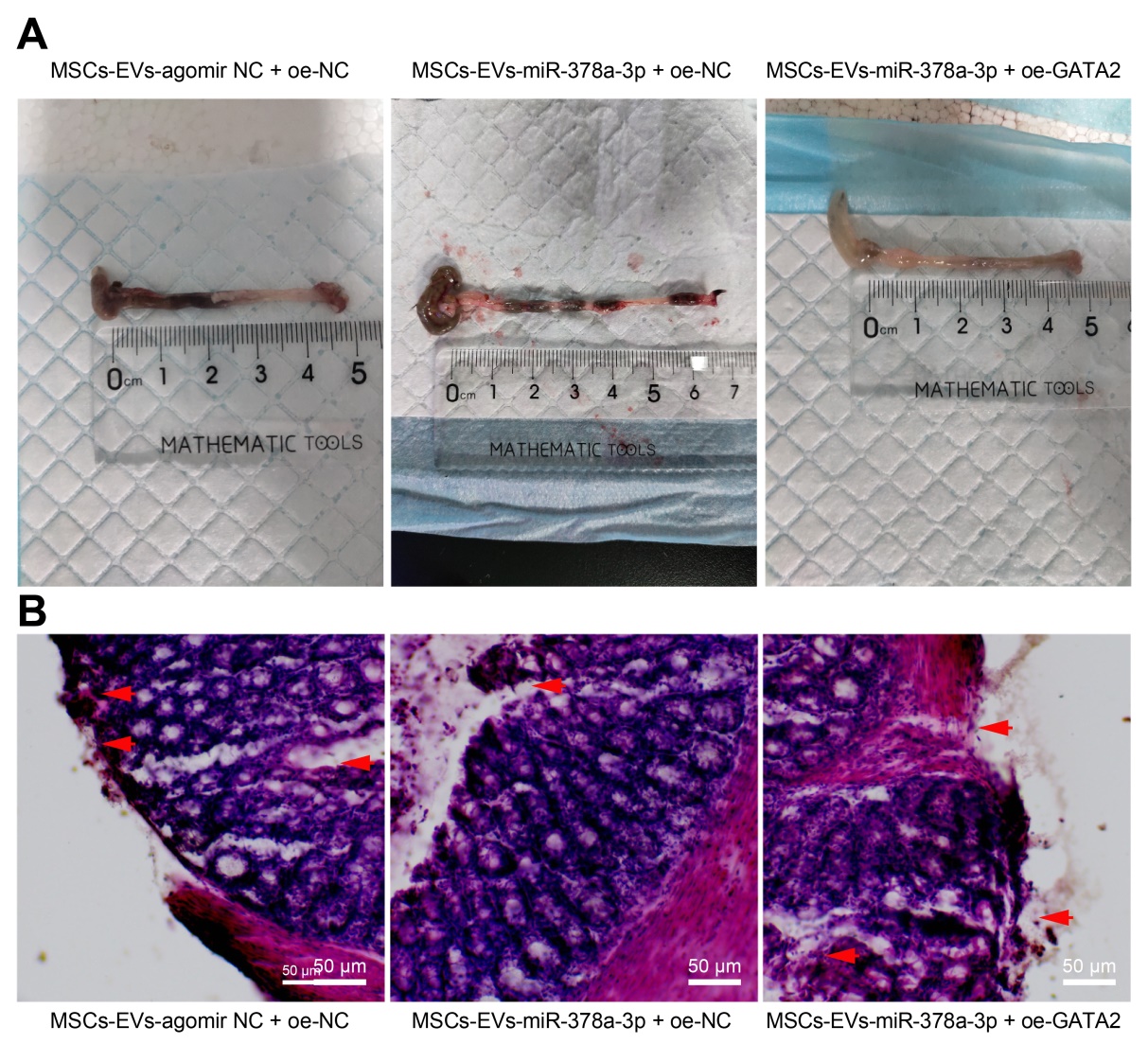
**

**FIGURE S3** A, Colon length of mice with different treatment was recorded. B, The statistical plot was drawn for colon tissue injury score of mice with different treatment as detected by HE staining (scale bar: 50 μm).
