# Supplementary material for: Mesenchymal stem cells‐derived extracellular vesicles containing miR‐378a‐3p inhibit the occurrence of inflammatory bowel disease by targeting GATA2
Source: J Cell Mol Med. 2022 May 17;26(11):3133–46. doi: 10.1111/jcmm.17176 (PMC9170824; doi:10.1111/jcmm.17176)
Supplement: Supplementary file 2 — Table S1‐S4 [file JCMM-26-3133-s001.docx]

**TABLE S1** Clinical characteristics of inflammatory bowel disease patients

|  | Biopsy samples | |
| --- | --- | --- |
|  | Healthy controls | IBD |
| Numbers of patients | 17 | 35 |
| Age (years) | 36.88 ± 7.24 | 41.00±8.93 |
| Gender |  |  |
| Male | 10 | 19 |
| Female | 7 | 16 |
| Disease duration (months) |  | 48.69±15.47 |
| Mayo Score |  | 7.03±1.46 |
| Disease extent |  |  |
| E1 |  | 4 |
| E2 |  | 23 |
| E3 |  | 10 |

**TABLE S2** Primer sequences for RT-qPCR

| Gene | Primer sequences |
| --- | --- |
| GATA2 (mouse) | F: 5’-GCCGGGAGTGTGTCAACTG-3’ |
|  | R: 5’-AGGTGGTGGTTGTCGTCTGA-3’ |
| AQP4 (mouse) | F: 5’-CTGGAGCCAGCATGAATCCAG-3’ |
|  | R: 5’-TTCTTCTCTTCTCCACGGTCA-3’ |
| PPAR-α (mouse) | F: 5’-CAGTGGGGAGAGAGGACAGA-3’ |
|  | R: 5’-AGTTCGGGAACAAGACGTTG-3’ |
| GAPDH (mouse) | F: 5’-ACATCATCCCTGCCTCTACTG-3’ |
|  | R: 5’-TTTGGCAGGTTTTTCTAGACGG-3’ |
| miR-378a-3p (mouse) | F: 5’-CGCGACTGGACTTGGAGTC-3’ |
|  | R: Universal reverse primer |
| U6 (mouse) | F: 5’-ACGAATACCGGCGTGAGAAA-3’ |
|  | R: Universal reverse primer |

Note: RT-qPCR, Reverse transcription-quantitative polymerase chain reaction; GATA2, GATA binding protein 2; AQP4, aquaporin-4; PPAR-α, peroxisome proliferator-activated receptor α; GAPDH, glyceraldehyde-phosphate dehydrogenase; miR-378a-3p, microRNA-378a-3p

**TABLE S3** DAI scoring system

| Score | Weight loss (%) | Stool character | Fecal occult blood |
| --- | --- | --- | --- |
| 0 | 0 | Normal formed | Negative |
| 1 | 1-5% |  |  |
| 2 | 5-10% | Loose stool | Positive |
| 3 | 10-20% |  |  |
| 4 | > 20% | Diarrhea | Gross bleeding |

Note: DAI, disease activity index

**TABLE S4** GATA2 binding domains in AQP4 promoter region

| Matrix ID | Name | Score | Relative score | Sequence ID | Start | End | Strand | Predicted sequence |
| --- | --- | --- | --- | --- | --- | --- | --- | --- |
| MA0036.1 | GATA2 | 6.65143 | 1.000000007 | AQP4 | 1447 | 1451 | - | GGATA |
| MA0036.1 | GATA2 | 6.23202 | 0.975650738 | AQP4 | 1659 | 1663 | - | AGATA |
| MA0036.1 | GATA2 | 5.9783 | 0.960920821 | AQP4 | 38 | 42 | - | GGATG |
| MA0036.1 | GATA2 | 5.9783 | 0.960920821 | AQP4 | 909 | 913 | - | GGATG |
| MA0036.1 | GATA2 | 5.9783 | 0.960920821 | AQP4 | 1411 | 1415 | - | GGATG |
| MA0036.1 | GATA2 | 5.77818 | 0.949302466 | AQP4 | 932 | 936 | - | TGATA |
| MA0036.1 | GATA2 | 5.77818 | 0.949302466 | AQP4 | 1421 | 1425 | + | TGATA |
| MA0036.1 | GATA2 | 5.55889 | 0.93657158 | AQP4 | 395 | 399 | - | AGATG |
| MA0036.1 | GATA2 | 5.55889 | 0.93657158 | AQP4 | 944 | 948 | + | AGATG |
| MA0036.1 | GATA2 | 5.55889 | 0.93657158 | AQP4 | 971 | 975 | + | AGATG |
| MA0036.1 | GATA2 | 5.55889 | 0.93657158 | AQP4 | 1126 | 1130 | + | AGATG |
| MA0036.1 | GATA2 | 5.10505 | 0.91022328 | AQP4 | 25 | 29 | + | TGATG |
| MA0036.1 | GATA2 | 5.10505 | 0.91022328 | AQP4 | 64 | 68 | - | TGATG |
| MA0036.1 | GATA2 | 5.10505 | 0.91022328 | AQP4 | 552 | 556 | - | TGATG |
| MA0036.1 | GATA2 | 5.10505 | 0.91022328 | AQP4 | 602 | 606 | + | TGATG |
| MA0036.1 | GATA2 | 5.10505 | 0.91022328 | AQP4 | 1043 | 1047 | + | TGATG |
| MA0036.1 | GATA2 | 5.10505 | 0.91022328 | AQP4 | 1132 | 1136 | + | TGATG |
| MA0036.1 | GATA2 | 5.04698 | 0.906851967 | AQP4 | 271 | 275 | + | GGATC |
| MA0036.3 | GATA2 | 9.65618 | 0.901468935 | AQP4 | 1443 | 1453 | + | TTCTTATCCCC |
| MA0036.1 | GATA2 | 4.87337 | 0.896772935 | AQP4 | 466 | 470 | - | GGATT |
| MA0036.1 | GATA2 | 4.87337 | 0.896772935 | AQP4 | 802 | 806 | + | GGATT |
| MA0036.1 | GATA2 | 4.87337 | 0.896772935 | AQP4 | 874 | 878 | - | GGATT |
| MA0036.1 | GATA2 | 4.87337 | 0.896772935 | AQP4 | 1083 | 1087 | - | GGATT |
| MA0036.1 | GATA2 | 4.87337 | 0.896772935 | AQP4 | 1682 | 1686 | + | GGATT |
| MA0036.1 | GATA2 | 4.87337 | 0.896772935 | AQP4 | 1799 | 1803 | - | GGATT |
| MA0036.3 | GATA2 | 8.80584 | 0.88401751 | AQP4 | 928 | 938 | + | TTATTATCATT |
| MA0036.1 | GATA2 | 4.62756 | 0.882502725 | AQP4 | 272 | 276 | - | AGATC |
| MA0036.1 | GATA2 | 4.62756 | 0.882502725 | AQP4 | 1098 | 1102 | + | AGATC |
| MA0036.1 | GATA2 | 4.62756 | 0.882502725 | AQP4 | 1099 | 1103 | - | AGATC |
| MA0036.1 | GATA2 | 4.62756 | 0.882502725 | AQP4 | 1603 | 1607 | + | AGATC |
| MA0036.3 | GATA2 | 8.24797 | 0.872568334 | AQP4 | 1519 | 1529 | + | TCCTTATTTTT |
| MA0036.1 | GATA2 | 4.45396 | 0.872423694 | AQP4 | 261 | 265 | + | AGATT |
| MA0036.1 | GATA2 | 4.45396 | 0.872423694 | AQP4 | 453 | 457 | - | AGATT |
| MA0036.1 | GATA2 | 4.45396 | 0.872423694 | AQP4 | 639 | 643 | - | AGATT |
| MA0036.1 | GATA2 | 4.45396 | 0.872423694 | AQP4 | 650 | 654 | - | AGATT |
| MA0036.1 | GATA2 | 4.45396 | 0.872423694 | AQP4 | 879 | 883 | - | AGATT |
| MA0036.1 | GATA2 | 4.45396 | 0.872423694 | AQP4 | 1275 | 1279 | - | AGATT |
| MA0036.1 | GATA2 | 4.45396 | 0.872423694 | AQP4 | 1454 | 1458 | + | AGATT |
| MA0036.1 | GATA2 | 4.45396 | 0.872423694 | AQP4 | 1788 | 1792 | + | AGATT |
| MA0036.1 | GATA2 | 4.45396 | 0.872423694 | AQP4 | 1928 | 1932 | + | CGATT |
| MA0036.1 | GATA2 | 4.45396 | 0.872423694 | AQP4 | 1947 | 1951 | - | AGATT |
| MA0036.1 | GATA2 | 4.45396 | 0.872423694 | AQP4 | 1996 | 2000 | - | AGATT |
| MA0036.2 | GATA2 | 8.20933 | 0.863520856 | AQP4 | 1440 | 1453 | + | TACTTCTTATCCCC |
| MA0036.3 | GATA2 | 7.59552 | 0.859178211 | AQP4 | 1015 | 1025 | - | CTCTTATTTTC |
| MA0036.1 | GATA2 | 4.17372 | 0.856154426 | AQP4 | 1604 | 1608 | - | TGATC |
| MA0036.1 | GATA2 | 4.17372 | 0.856154426 | AQP4 | 1884 | 1888 | + | TGATC |
| MA0036.1 | GATA2 | 4.17372 | 0.856154426 | AQP4 | 1885 | 1889 | - | TGATC |
| MA0036.2 | GATA2 | 7.53954 | 0.854389092 | AQP4 | 821 | 834 | + | TCTTTCTTCTCTCA |
| MA0036.2 | GATA2 | 7.21894 | 0.850018054 | AQP4 | 1592 | 1605 | + | TGCTTCTTCTCAGA |
| MA0036.1 | GATA2 | 4.00011 | 0.846075394 | AQP4 | 491 | 495 | - | TGATT |
| MA0036.1 | GATA2 | 4.00011 | 0.846075394 | AQP4 | 1061 | 1065 | - | TGATT |
| MA0036.1 | GATA2 | 4.00011 | 0.846075394 | AQP4 | 1073 | 1077 | + | TGATT |
| MA0036.1 | GATA2 | 4.00011 | 0.846075394 | AQP4 | 1347 | 1351 | - | TGATT |
| MA0036.1 | GATA2 | 4.00011 | 0.846075394 | AQP4 | 1538 | 1542 | - | TGATT |
| MA0036.1 | GATA2 | 4.00011 | 0.846075394 | AQP4 | 1978 | 1982 | - | TGATT |
| MA0036.2 | GATA2 | 6.48297 | 0.839984022 | AQP4 | 1893 | 1906 | - | AGTAGCTTTTCTGT |
| MA0036.2 | GATA2 | 6.19727 | 0.8360888 | AQP4 | 1601 | 1614 | - | AGTTTTTGATCTGA |
| MA0036.2 | GATA2 | 6.16965 | 0.835712185 | AQP4 | 925 | 938 | + | GAGTTATTATCATT |
| MA0036.1 | GATA2 | 3.78256 | 0.83344497 | AQP4 | 580 | 584 | + | GCATA |
| MA0036.1 | GATA2 | 3.78256 | 0.83344497 | AQP4 | 1259 | 1263 | - | GCATA |
| MA0036.2 | GATA2 | 5.37605 | 0.824892467 | AQP4 | 1882 | 1895 | - | TGTACCTGATCACT |
| MA0036.2 | GATA2 | 5.36018 | 0.82467613 | AQP4 | 509 | 522 | + | TAAGTGTTCTCTGT |
| MA0036.2 | GATA2 | 5.15667 | 0.821901446 | AQP4 | 1780 | 1793 | - | AAATCTTTATTTGA |
| MA0036.2 | GATA2 | 5.15058 | 0.821818466 | AQP4 | 872 | 885 | + | ATAATCCAATCTGA |
| MA0036.3 | GATA2 | 5.75836 | 0.821474167 | AQP4 | 325 | 335 | + | GCCTTAGCTTC |
| MA0036.3 | GATA2 | 5.7156 | 0.820596558 | AQP4 | 1893 | 1903 | - | AGCTTTTCTGT |
| MA0036.3 | GATA2 | 5.4832 | 0.815827055 | AQP4 | 824 | 834 | + | TTCTTCTCTCA |
| MA0036.2 | GATA2 | 4.51827 | 0.81319761 | AQP4 | 841 | 854 | + | TCTTTCTTGTCATA |
| MA0036.2 | GATA2 | 4.50156 | 0.812969766 | AQP4 | 1667 | 1680 | + | TTCATGTTAACTGC |
| MA0036.2 | GATA2 | 4.49065 | 0.812821047 | AQP4 | 1652 | 1665 | + | AAGTCTCTATCTAA |
| MA0036.2 | GATA2 | 4.28872 | 0.810067979 | AQP4 | 652 | 665 | + | TCTTGGTTAGCTGA |
| MA0036.1 | GATA2 | 3.36314 | 0.809095715 | AQP4 | 626 | 630 | + | CCATA |
| MA0036.1 | GATA2 | 3.36314 | 0.809095715 | AQP4 | 1028 | 1032 | + | CCATA |
| MA0036.1 | GATA2 | 3.36314 | 0.809095715 | AQP4 | 1396 | 1400 | + | CCATA |
| MA0036.1 | GATA2 | 3.36314 | 0.809095715 | AQP4 | 1513 | 1517 | - | ACATA |
| MA0036.1 | GATA2 | 3.36314 | 0.809095715 | AQP4 | 1728 | 1732 | + | ACATA |
| MA0036.3 | GATA2 | 5.12325 | 0.808439964 | AQP4 | 1113 | 1123 | - | TTATTATTTTT |
| MA0036.2 | GATA2 | 4.12275 | 0.807805198 | AQP4 | 1516 | 1529 | + | GTTTCCTTATTTTT |
| MA0036.2 | GATA2 | 3.78404 | 0.803187317 | AQP4 | 1728 | 1741 | - | GTTTGGTTATATGT |
| MA0036.3 | GATA2 | 4.7508 | 0.800796047 | AQP4 | 1655 | 1665 | + | TCTCTATCTAA |
| MA0036.3 | GATA2 | 4.73226 | 0.800415563 | AQP4 | 1780 | 1790 | - | TCTTTATTTGA |
